# Aldehyde Dehydrogenase 2 Ameliorates LPS-Induced Acute Kidney Injury through Detoxification of 4-HNE and Suppression of the MAPK Pathway

**DOI:** 10.1155/2023/5513507

**Published:** 2023-04-06

**Authors:** Jifu Jin, Rebecca Suchi Chang, Sujuan Xu, Guang Xia, Jennifer Ming Jen Wong, Yi Fang, Ping Jia, Xiaoqiang Ding

**Affiliations:** ^1^Department of Cardiology, Shanghai East Hospital, School of Medicine, Tongji University, Shanghai, China; ^2^Department of Nephrology, Zhongshan Hospital, Fudan University, Shanghai, China; ^3^Department of Cardiology, Zhongshan Hospital, Shanghai Institute of Cardiovascular Diseases, Fudan University, Shanghai, China; ^4^National Clinical Research Center for Interventional Medicine, Shanghai, China; ^5^Orthopedic Research Institute of Hebei Province, Third Hospital of Hebei Medical University, Shijiazhuang, China; ^6^Department of Nephrology, Third Hospital of Hebei Medical University, Shijiazhuang, China

## Abstract

Lipopolysaccharide (LPS)-induced septic acute kidney injury (AKI) is determined as a devastating organ dysfunction elicited by an inappropriate response to infection with high morbidity and mortality rates. Previous evidence has illustrated an indispensable role of mitochondrial aldehyde dehydrogenase 2 (ALDH2) in the pathogenesis of sepsis-induced multiorgan abnormalities. Specifically, this study investigated the potential role of ALDH2 in sepsis-induced AKI. After LPS administration, we observed a significant decline in renal function, increased inflammatory cytokines, oxidative stress, 4-hydroxy-2-nonenal (4-HNE) accumulation, and apoptosis via MAPK activation in ALDH2^−/−^ mice; in contrast, pretreatment with Alda-1 (an ALDH2 activator) alleviated the LPS-induced dysfunctions in mice. Moreover, in vitro analysis revealed that ALDH2 overexpression in mouse tubular epithelial cells (mTECs) improved the inflammatory response, oxidative stress, 4-HNE accumulation, and apoptosis via MAPK inhibition, whereas ALDH2 knockdown in mTECs aggravated these parameters via MAPK activation. Therefore, ALDH2 may protect against LPS-induced septic AKI by suppressing 4-HNE/MAPK pathway.

## 1. Introduction

Sepsis is a life-threatening organ dysfunction, presenting as a systemic inflammatory response syndrome with a high mortality rate of up to 15%–20% [1]. Septic acute kidney injury (AKI), one of the leading causes of AKI in critically ill patients, accounts for 45%–70% of AKI cases in the intensive care unit settings [2]. The short-term mortality rate of septic AKI is 50% higher than that of nonseptic AKI in patients with septic shock [3]. Current therapies for septic AKI are limited to supportive options, including antibiotic uses, fluid resuscitation, and early hemodynamic optimization; however, the morbidity and mortality of septic AKI remain high. Although recent studies have illustrated that the pathophysiology of septic AKI involves inflammation, microvascular dysfunction, oxidative stress, and apoptosis, the precise mechanisms remain poorly understood [4, 5].

Aldehyde dehydrogenase 2 (ALDH2) is an essential mitochondrial enzyme within the ALDH family that has the highest affinity for the detoxification of reactive aldehydes, including acetaldehyde and 4-hydroxy-2-nonenal (4-HNE) [6]. Genetic polymorphisms of ALDH2 exist in ∼40% of the Asian population with severely compromised enzymatic activity [7]. Previous studies have depicted a pivotal role for ALDH2 in various diseases, including myocardial ischemia-reperfusion injury, heart failure, diabetic cardiomyopathy, ischemic stroke, atherosclerosis, and alcoholic liver disease via the regulation of inflammation, oxidative stress, pyroptosis, and autophagy [8–13]. Moreover, recent studies have shed light on the protective role of ALDH2 in sepsis-induced organ dysfunction [14–18]. The activation of ALDH2 may alleviate sepsis-induced cardiac dysfunction or acute lung injury severity by modulating endoplasmic reticulum stress, autophagy, and inflammation [15–17].

A preliminary study reported that the inhibition of ALDH2 expression in the kidneys aggravated renal injury and inflammation in a sepsis model [18]. After inhibition of ALDH2 expression with cyanamide, the mean arterial blood pressure was decreased, and the glomerular atrophy was further aggravated [18]. Moreover, plasma creatinine, urea nitrogen, malondialdehyde, and nuclear factor kappa B (NF-*κ*B) levels increased significantly after LPS injection when compared to the vehicle-treated group [18]. Nevertheless, the potential effect of ALDH2 on the pathogenesis of septic AKI and related signal pathways need to be elucidated.

Therefore, in the present study, we hypothesized that ALDH2 plays an essential role in sepsis-induced AKI via the inhibition of inflammation, oxidative stress, and apoptosis. Moreover, combined with *in vivo* and *in vitro* experiments, we attempted to clarify the potential molecular pathway underlying the protective role of ALDH2 against septic AKI.

## 2. Materials and Methods

### 2.1. Animal Models

Eight to ten weeks old C57BL/6 (wild-type (WT)) mice, ALDH2^−/−^ mice, and their littermates weighing 20–25 g were used in our experiments. The C57BL/6 mice were obtained from the Shanghai Animal Administration Center (Shanghai, China), and the ALDH2^−/−^ mice were provided by Professor Sun (Fudan University). All the mice were housed in a climate-controlled environment with free access to food and water *ad libitum*. The sepsis model was induced via intraperitoneal (IP) injection of lipopolysaccharide (LPS) from *Escherichia coli* O55:B5 (Sigma, St. Louis, MO, USA) at a dose of 10 mg/kg. Alda-1 (Sigma, St. Louis, MO, USA), an ALDH2-specific activator, was administered via IP injection at a dose of 10 mg/kg 1 hr before LPS administration. An IP injection of an equal volume of saline or DMSO (Sigma) served as vehicle-treated groups. The mice were randomized into the following groups: WT, ALDH2 knockout (KO), WT + LPS (10 mg/kg, IP), KO + LPS (10 mg/kg, IP), WT + Alda-1 (10 mg/kg, IP), and WT + Alda-1 + LPS (10 mg/kg, respectively, IP). All animal study protocols were approved by the Animal Care and Use Committee of Fudan University (Shanghai, China). All animal procedures followed the National Institutes of Health's guidelines for the care and use of laboratory animals.

### 2.2. Cell Culture and Lentivirus Transfection

Mouse renal tubular epithelial cells (mTECs) were obtained from Caltag MedSystems (Buckingham, UK). The cells were cultured in Dulbecco's modified Eagle's medium supplemented with 100 U/mL penicillin, 100 *μ*g/mL streptomycin, and 10% fetal bovine serum at 37°C in humidified 5% CO_2_. ALDH2 overexpression (OE), ALDH2 knockdown (KD), and control lentivirus (NC) vectors (Hanyin, Shanghai, China) were used to deliver ALDH2 OE or inhibition respectively to mTECs conforming to the manufacturer's protocols. The successful modification of ALDH2 protein levels was confirmed using western blot analysis. To assess the role of ALDH2 in LPS-induced TEC dysfunction, ALDH2-OE, ALDH2-KD, and NC cells were exposed to 1 or 10 *μ*g/mL LPS (Sigma) for 24 hr before biochemical analysis. In addition, treatment with 10 or 20 *μ*M of the ALDH2 activator Alda-1 (Sigma) before LPS (1 *μ*g/mL) was employed to demonstrate the protective effect of ALDH2 on LPS-induced mTECs dysfunction.

### 2.3. Serum Creatinine Measurement

According to the manufacturer's protocols, serum creatinine levels in mice were evaluated using the QuantiChromTM Creatinine Assay Kit (Bioassay Systems, Hayward, CA, USA).

### 2.4. Cytokine Analysis

Serum levels of mouse tumor necrosis factor-*α* (TNF-*α*) and interlukin-6 (IL-6) were detected using an enzyme-linked immunosorbent assay (ELISA) kit (ABclonal, Wuhan, China), according to the manufacturer's instructions.

### 2.5. Reactive Oxygen Species (ROS) Measurement

ROS measurements of kidney tissue from frozen sections and TECs loaded onto 96-well plates were incubated through dihydroethidium staining (Sigma), conforming to the manufacturer's instructions. Nuclei were incubated with 4′,6-diamidino-2-phenylindole (DAPI). Images were acquired using an FSX-100 microscope (Olympus, Tokyo, Japan) and quantified using ImageJ software.

### 2.6. Terminal Deoxynucleotidyl Transferase dUTP Nick end Labeling (TUNEL) Assay

Apoptosis in kidney tissue or TECs was analyzed using a commercially available TUNEL assay kit (Beyotime Biotechnology, Haimen, China), conforming to the manufacturer's protocols. The nuclei were stained with DAPI. The number of TUNEL-positive cells in random areas was counted using an FSX-100 microscope (Olympus).

### 2.7. ALDH2 Activity Measurement

ALDH2 activity was detected in 33 mM sodium pyrophosphate, including 0.8 mM nicotinamide adenine dinucleotide (NAD^+^), 15 *μ*M propionaldehyde, as well as 0.1 mL kidney tissue extraction. Specifically, ALDH2 activity was determined with the reduction of NAD^+^ to NADH, along with the oxidization of propionaldehyde to propionic acid. The NADH concentration was estimated spectrophotometrically at an absorbance wavelength of 340 nm.

### 2.8. Histology and Immunohistochemistry

Formalin-fixed kidney tissues were embedded in paraffin and cut into sections for hematoxylin and eosin staining. For immunochemistry staining, kidney tissues were incubated with primary antibodies (4-HNE and NF-*κ*B) at 4°C overnight, washed with phosphate-buffered saline (PBS), and incubated with horseradish peroxidase (HRP)-conjugated anti-rabbit IgG secondary antibody for 1 hr at 26°C environment. Finally, DAPI was utilized to counterstain the nuclei. The stained sections were analyzed and photographed using an FSX-100 microscope (Olympus).

### 2.9. Flow Cytometry

Apoptotic TECs were estimated by flow cytometry using the Annexin V-fluorescein isothiocyanate (FITC)/propidium iodide (PI) Kit (Sigma–Aldrich), in accordance with the manufacturer's instructions. TECs stained with Annexin V (FITC) or PI were determined using flow cytometry (Beckman Coulter, Brea, CA, USA) and analyzed using the FlowJo software (BD, OR, USA).

### 2.10. Quantitative Real-Time Polymerase Chain Reaction (RT-PCR)

Total RNA was extracted from kidney tissues and TECs using TRIzol Reagent (Sigma–Aldrich, T9424-200 mL). cDNA was synthesized from the RNA using a PrimeScript RT Reagent Kit (TaKaRa, Shiga, Japan), conforming to the manufacturer's protocols. A 10 *μ*L mixture of the diluted cDNA and a variety of gene-specific primers was incubated with SYBR Premix Ex Taq (TaKaRa) and then subjected to RT-PCR quantification using an RT-PCR System (Applied Biosystems, Waltham, MA, USA). The primer sequences used were as follows (Table 1).

### 2.11. Western Blot Analysis

Proteins were extracted from kidney tissue or TECs using RIPA lysis buffer (Beyotime Biotechnology, China). Aliquots of the protein samples mixed with loading buffer were then separated by 10%–15% sodium dodecyl sulfate polyacrylamide gel electrophoresis. The proteins were then electrically transferred to polyvinylidene difluoride membranes (Millipore, Burlington, MA, USA). The membranes were immersed in the nonfat skim milk for 1 hr prior to the incubation with primary antibodies at 4°C overnight. The primary antibodies used in the study were as follows: anti-*β*-actin (Wei'ao, Shanghai, China; 1 : 5,000), anti-ALDH2 (Abcam, Cambridge, UK; 1 : 3,000), anti-4-HNE (Abcam, Cambridge, UK; 1 : 3,000), anti-pP38 (CST, MA, USA; 1 : 3,000), anti-P38 (CST, MA, USA; 1 : 3,000), anti-pJNK (CST, MA, USA; 1 : 3,000), anti-JNK (CST, MA, USA; 1 : 3,000), anti-pNF-*κ*B (CST, MA, USA; 1 : 1,000), anti-NF-*κ*B (CST, MA, USA; 1 : 1,000), anti-Bax (CST, MA, USA; 1 : 3,000), anti-Bcl-2 (CST, MA, USA; 1 : 3,000), and anti-cleaved caspase-3 (CST, MA, USA; 1 : 1,000). HRP-conjugated secondary antibodies (Kangcheng, Shanghai, China) were used to the incubation of membranes after washing with PBS containing Tween-20 (PBST). Detection of bound antibody was carried out with the chemiluminescence system (Thermo Fisher Scientific, Waltham, MA, USA). Protein expression levels were quantified using Image J software. Specifically, background subtraction was performed using Volume Box Tools in the software. Next, western blot data were normalized with housekeeping protein *β*-actin. After obtaining normalized values, each condition was performed in triplicate for further quantitative analysis.

### 2.12. Statistical Analysis

Statistical analysis was conducted using SPSS software and GraphPad Prism. Continuous data are presented as mean ± SEM for all analyses. One-way analysis of variance with the multiple comparisons test (Tukey or Bonferroni test) was utilized for analysis of intergroup differences. A *P* significance was set at *P* < 0.05.

## 3. Results

### 3.1. ALDH2 Expression and Activity in Kidneys after LPS Administration

**Figure 1:**
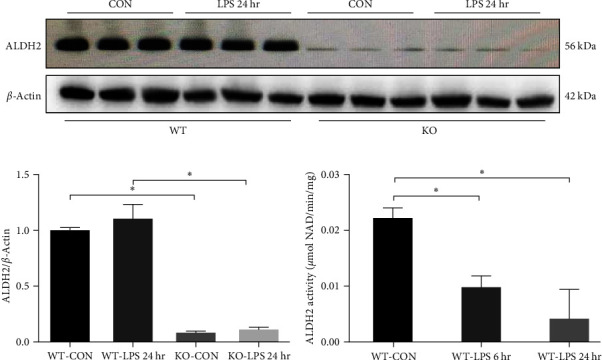
ALDH2 expression in kidneys at 24 hr after LPS treatment in wild-type (WT) and ALDH2 knockout (KO) mice and ALDH2 activity at 6 and 24 hr after LPS treatment in WT mice; (a, b) representative western blots and quantification analysis of ALDH2 and *β*-actin (loading control); (c) quantification of ALDH2 enzymatic activity in kidneys at 6 and 24 hr after LPS treatment in WT mice. All data are shown as mean ± SEM (*n* = 6).  ^*∗*^*P* < 0.05.

The ALDH2 protein expression of WT and KO mice and the protein activity in WT mice were analyzed via western blot and ALDH2 activity detection kit, respectively, with or without LPS challenge. The protein levels of ALDH2 in the kidneys were retained at 24 hr of LPS administration, whereas the activity of ALDH2 was significantly inhibited at 6 hr of LPS administration and further declined at 24 hr of LPS treatment when compared to the vehicle-treated WT group (Figure 1(a)–1(c)). In addition, western blot analysis showed that ALDH2 protein level was drastically decreased in the kidneys of KO mice than in those of WT mice (Figures 1(a) and 1(b)).

### 3.2. Effect of ALDH2 Knockout or Activation on LPS-Induced Acute Tubular Injury and Renal Function

**Figure 2:**
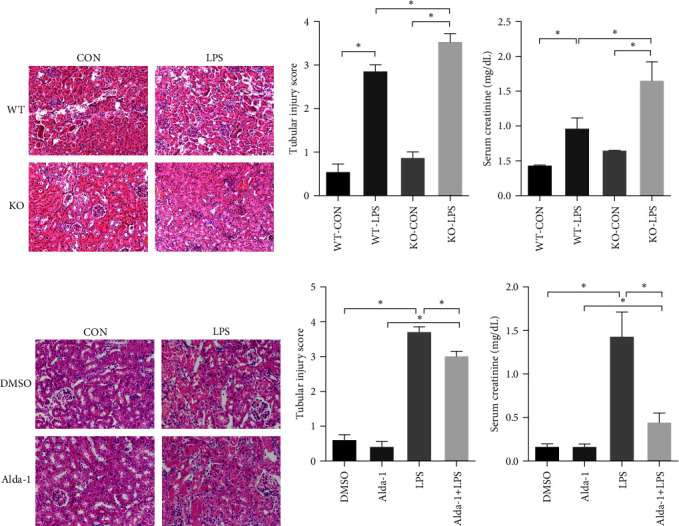
Effect of ALDH2-KO or ALDH2 activator Alda-1 pretreatment on the LPS-induced acute tubular injury and increase of serum creatinine (SCr). The characteristic of tubular injury included tubular epithelia swelling, loss of brush border, vacuolar degenerating, and tubular forming. The severity of tubular injury score was defined from 0 to 4 according to the percentage of injured area: 0, normal tissue; 1, damaged area <25%; 2, damaged area of 25%–50%; 3, damaged are of 50%–75%; 4, damaged area of 75%–100%: (a) the collected kidneys of WT and KO mice were stained with hematoxylin and eosin (H&E); (b) quantitative evaluation of morphological tubular damage in WT and KO mice; (c) quantification of SCr in WT and KO mice; (d) the collected kidneys of mice with or without pretreatment of Alda-1 were stained with H&E; (e) quantitative evaluation of morphological tubular damage in mice with or without Alda-1 pretreatment; (f) quantification of SCr in mice with or without Alda-1 pretreatment. Scale bars: 50 *μ*m. All data are expressed as mean ± SEM (*n* = 6).  ^*∗*^*P* < 0.05.

Histological injury occurred in the kidneys 24 hr at LPS administration and was characterized by tubular cell degeneration, loss of brush border, and inflammatory cell infiltration. In addition, morphological damage was assessed by histopathological scoring of the kidneys. Interestingly, morphological damage and histopathological scoring were increased in the kidneys of KO mice compared to that in WT mice (Figures 2(a) and 2(b)). Additionally, KO mice showed a significant increase in serum creatinine levels after LPS injection compared with WT mice (Figure 2(c)).

Similarly, pretreatment with Alda-1, an ALDH2 selective agonist, attenuated the morphological damage and histopathological scoring of the kidneys after LPS administration compared with that of the vehicle-treated WT mice (Figures 2(d) and 2(e)). Mice pretreated with Alda-1 also showed a significant decrease in serum creatinine levels when compared to the vehicle-treated group after LPS injection, indicating that there is an inverse relationship between ALDH2 activity and serum creatinine levels (Figure 2(f)).

### 3.3. ALDH2 Regulated the LPS-Induced Inflammatory Response and NF-*κ*B Activation

**Figure 3:**
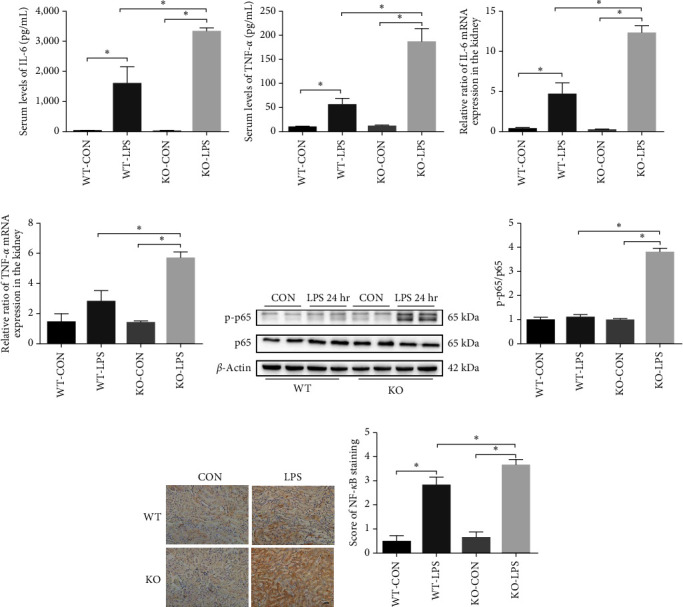
Role of ALDH2 on the LPS-induced inflammatory response and NF-*κ*B activation: (a, b) protein levels of interleukin (IL)-6 and tumor necrosis factor (TNF)-*α* in the kidney homogenate were measured using ELISA; (c, d) mRNA levels of IL-6 and TNF-*α* were measured by PCR analysis in the kidney homogenate; (e, f) representative western blots and quantification analysis of NF-*κ*B expression and *β*-actin; (g) immunohistochemical staining for NF-*κ*B expression in kidneys in WT and KO mice after LPS administration; (h) quantification of NF-*κ*B positive cells per field. Scale bars: 50 *μ*m. All data are expressed as mean ± SEM (*n* = 6).  ^*∗*^*P* < 0.05.

To explore the effect of ALDH2 on the inflammatory response, we examined multiple cytokines in the circulation and kidneys 24 hr after LPS administration. In the serum, the concentration of inflammatory cytokines TNF-*α* and IL-6 significantly increased 24 hr after the LPS injection in WT mice. Moreover, the serum TNF-*α* and IL-6 levels in KO mice were significantly higher than those in WT mice (Figures 3(a) and 3(b)). In addition, quantitative real-time reverse transcription-PCR analysis indicated that mRNA expression levels of TNF-*α* and IL-6 in the kidneys of KO mice were drastically increased compared to those in WT mice (Figures 3(c) and 3(d)).

Additionally, we evaluated the effects of Alda-1 on systemic and renal inflammation. We found that pretreatment with Alda-1 significantly decreased the concentration of serum proinflammatory cytokines (TNF-*α* and IL-6) compared with that in the vehicle-treated group (Supplementary Figures S1(a) and S1(b)). Furthermore, in accordance with the protein levels, the mRNA levels of TNF-*α* and IL-1*β* were also significantly downregulated in mice pretreated with Alda-1 after LPS administration (Supplementary Figures S1(c) and S1(d)).

NF-*κ*B is an important transcription factor downstream of the endotoxin signaling pathway [19]. To further investigate the effect of ALDH2 deficiency on NF-*κ*B expression, NF-*κ*B p65 levels were detected by western blotting and immunohistochemistry. These results indicated that the phosphorylation levels of NF-*κ*B p65 in the kidneys of KO mice were remarkably upregulated compared to that in WT mice (Figure 3(e)–3(h)).

### 3.4. ALDH2 Regulated the LPS-Induced 4-HNE Accumulation and ROS Generation

**Figure 4:**
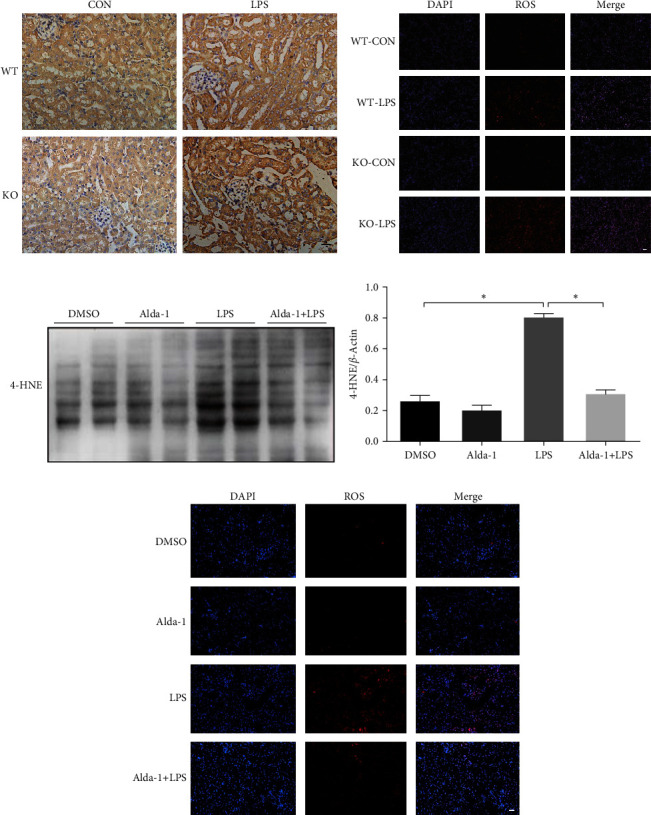
Role of ALDH2 on the LPS-induced 4-HNE accumulation and ROS generation: (a) immunohistochemical staining for 4-HNE expression in kidneys in WT and KO mice after LPS administration; (b) ROS expression in kidneys in WT and KO mice after LPS administration; (c, d) representative western blots and quantification analysis of 4-HNE and *β*-actin (loading control) with or without Alda-1 pretreatment in WT mice; (e) ROS expression in kidneys with or without Alda-1 pretreatment in WT mice. Scale bars: 50 *μ*m. All data are expressed as mean ± SEM (*n* = 6).  ^*∗*^*P* < 0.05.

4-HNE is one of the most toxic aldehydes produced during lipid peroxidation [20, 21]. To elucidate the potential mechanism of ALDH2's effect on the LPS-induced AKI, the lipid peroxidation end-product 4-HNE, a key ALDH2 substrate, and ROS, a major oxidative stress marker, were evaluated in ALDH2 KO and Alda-1 pretreatment mice, respectively. Our results indicated that LPS enhanced 4-HNE accumulation and ROS generation 24 hr after LPS injection, the effect of which was significantly augmented and attenuated by ALDH2 KO and Alda-1 pretreatment, respectively (Figure 4(a)–4(e)).

To confirm the protective effect of ALDH2 against LPS-induced tubular injury *in vitro*, the expression level of ALDH2 in mTECs was modified by infection with ALDH2 OE and KD lentivirus vectors or control lentivirus vectors (NC). The protein expression of ALDH2 in OE cells was significantly higher than that in NC cells, whereas that in KD cells was remarkably reduced. In line with *in vivo* study, ALDH2 expression in mTECs was retained 24 hr after LPS (1 *μ*g/mL) treatment (Supplementary Figure S2(a)–S2(c)). The *in vitro* study further demonstrated that 4-HNE accumulation and ROS generation were upregulated 24 hr after LPS treatment (1 or 10 *μ*g/mL), the effects of which were mitigated and augmented by ALDH2 OE and KD, respectively (Supplementary Figures S2(a)–S2(e) and S3(a) and S3(b)).

### 3.5. Effect of ALDH2 on LPS-Induced Apoptosis

**Figure 5:**
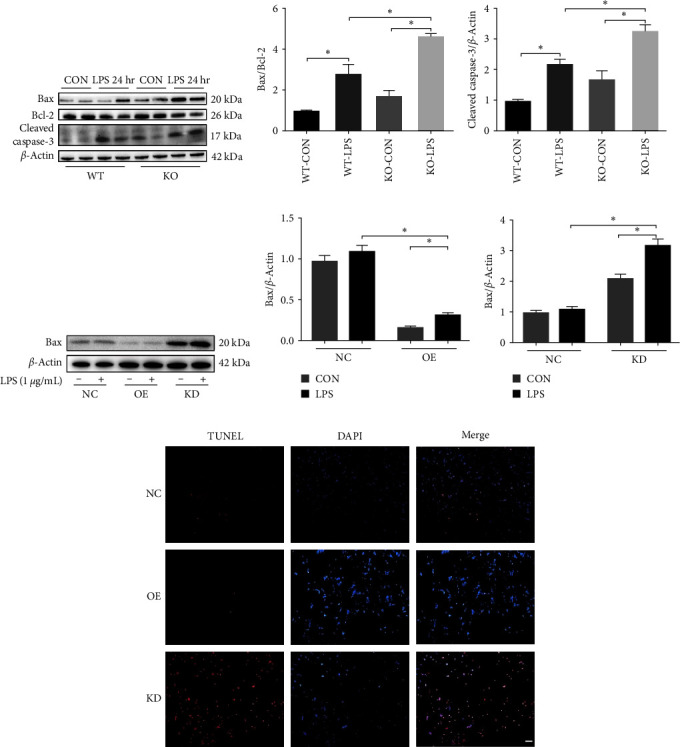
Effect of ALDH2 on LPS-induced apoptosis: (a–c) representative western blots and quantification analysis of Bax, Bcl2, cleaved caspase-3, and *β*-actin (loading control) in WT and KO mice after LPS administration; (d–f) representative western blots and quantification analysis of Bax and *β*-actin (loading control) in OE, KD, and NC cells after LPS challenging; (g) representative images of TUNEL assay in OE, KD, and NC cells after LPS challenging. Scale bars: 50 *μ*m. All data are expressed as mean ± SEM (*n* = 6).  ^*∗*^*P* < 0.05.

To further validate the effect of ALDH2 on LPS-induced renal epithelial cell apoptosis, TUNEL staining, and western blotting were used to analyze the apoptotic cells in the kidneys. The results indicated that the protein expression of Bax and activity of caspase-3 were increased, whereas Bcl-2 was reduced in the kidneys after LPS injection. Furthermore, ALDH2 deficiency caused a significant increase in the expression of proapoptotic Bax and the activity of caspase-3 while causing a decrease in antiapoptotic Bcl-2 in the kidneys (Figure 5(a)–5(c)). In line with these results and in comparison with the vehicle-treated group, mice receiving Alda-1 pretreatment showed a significant reduction in the expression of proapoptotic Bax in the kidneys (Supplementary Figures S4(a) and S4(b)).

Additionally, the *in vitro* studies revealed that ALDH2 OE in TECs profoundly suppressed the expression of Bax and TUNEL-positive cells after LPS stimulation. However, silencing of ALDH2 in mTECs significantly increased the expression of Bax and TUNEL-positive cells compared to the control cells (Figure 5(d)–5(g)). Therefore, to further validate the antiapoptotic effect of ALDH2 on mTECs induced by LPS, apoptosis-related protein, TUNEL, and annexin V-FITC/PI staining were evaluated in the presence of Alda-1 (10 or 20 *μ*M). Our results indicated that the relative expression levels of Bax and Bcl-2 were slightly increased by pretreatment with low-dose Alda-1 (10 *μ*M); in contrast, they were significantly reduced by pretreatment with high-dose (20 *μ*M) Alda-1 (Supplementary Figures S5(a) and S5(b)). Likewise, high-dose Alda-1 (20 *μ*M) pretreatment rescued TUNEL-positive cells and late apoptotic cells by flow cytometric analysis after LPS induction (Supplementary Figure S5(c)–S5(e)).

### 3.6. ALDH2 Regulated the Apoptosis after LPS Administration via MAPK Signaling Pathway

**Figure 6:**
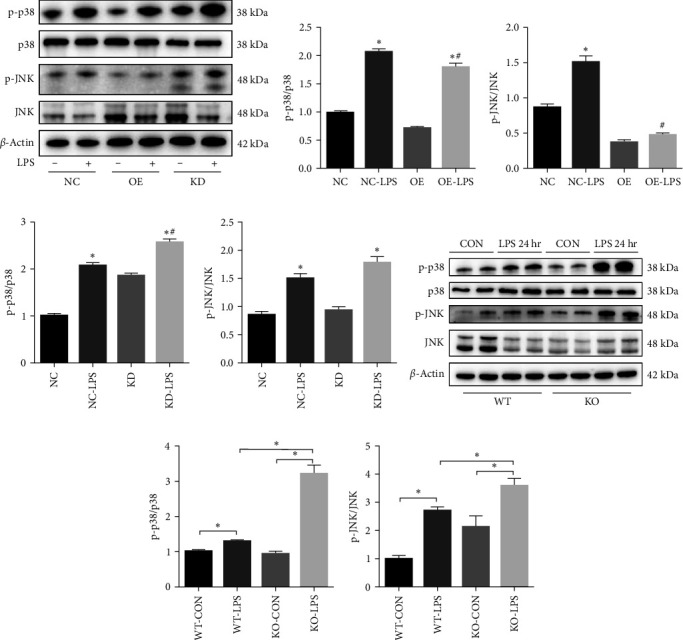
ALDH2 regulates MAPK signaling pathway after LPS administration: (a) representative western blots of p-p38, p38, p-JNK, JNK, and *β*-actin (loading control) in ALDH2 overexpression (OE), knockdown (KD), and control (NC) tubular epithelial cells (TECs) after LPS challenging; (b, c) quantification analysis of p-p38, p38, p-JNK, and JNK expression in OE *vs*. NC cells; (d, e) quantification analysis of p-p38, p38, p-JNK, and JNK expression in KD *vs*. NC cells; (f–h) representative western blots and quantification analysis of p-p38, p38, p-JNK, JNK, and *β*-actin (loading control) in WT and KO mice after LPS administration. All data are expressed as mean ± SEM (*n* = 6).  ^*∗*^*P* < 0.05*vs*. NC or OE group. ^#^*P* < 0.05*vs*. NC LPS group.

Previous studies have shown that 4-HNE profoundly activates the MAPK signaling pathway in cardiomyocytes [22]. Moreover, the MAPK signaling pathway plays an important role in regulating inflammation, apoptosis, and cell cycle arrest [23–26]. To determine whether ALDH2 regulated the MAPK signaling pathway after LPS challenge, ALDH2 OE or KD TECs were treated with LPS (1 *μ*g/mL), and MAPK signaling, JNK, and p38 expression were detected. Western blot analysis demonstrated that phosphorylation of p38 and JNK in TECs was upregulated 24 hr after LPS stimulation (1 *μ*g/mL). Interestingly, the OE of ALDH2 significantly attenuated the phosphorylation of p38 and JNK compared to that in the control (Figure 6(a)–6(c)). Additionally, the KD of ALDH2 profoundly increased the phosphorylation of p38, but not JNK, compared to that in the control (Figures 6(a), 6(d), and 6(e)). Similarly, the *in vivo* study revealed that the phosphorylation levels of p38 and JNK were significantly augmented in ALDH2 KO mice compared to that in controls (Figure 6(f)–6(h)).

## 4. Discussion

Sepsis is a detrimental clinical syndrome characterized by a dysregulated host response to infection [27]. LPS is the prime component of the outer membrane of Gram-negative bacteria and is one of the important means for the simulation of sepsis-induced organ dysfunction [28]. Previous studies have illustrated that ALDH2 protects against sepsis-induced multiorgan abnormalities [14–18]. Considering that ALDH2 protects cells from the effects of ROS and those of reactive nitrogen species, such as 4-HNE, we hypothesized that the activation of ALDH2 could exert a protective role against septic AKI. Therefore, we established a murine LPS-induced sepsis model to investigate the potential role and underlying mechanisms of ALDH2 in the pathogenesis of septic AKI. The results of *in vivo* and *in vitro* analyses together revealed that the protein levels of ALDH2 in the kidneys were consistent, whereas ALDH2 activity was remarkably reduced after LPS stimulation. We further examined the kidney injury score and the deterioration in renal function in ALDH2 KO mice in comparison with those observed in ALDH2 WT mice after the LPS challenge. Moreover, the levels of inflammatory cytokines (including TNF-*α* and IL-6) and NF-*κ*B p65 increased after LPS administration and were further augmented in ALDH2 KO mice compared with ALDH2 WT mice. Similarly, the levels of ROS and 4-HNE were elevated in ALDH2 KO mice after LPS treatment, which is consistent with previous reports [17, 29]. These results suggest that defects in ALDH2 intensify the decline in renal function as well as the inflammatory response and oxidative stress in LPS-induced septic AKI.

Mitochondrial function is crucial for energy metabolism and ROS production [30–32]. Disturbed mitochondrial electron transfer chains commonly generate ROS in response to an exacerbated inflammatory reaction during sepsis development [33, 34]. Considering that ALDH2 is an essential enzyme of respiratory chain in mitochondria, studies have demonstrated that ALDH2 deficiency generally induces ROS generation, whereas ALDH2 activation alleviates ROS accumulation in various diseases (including sepsis) [17, 35, 36]. Increased ROS levels further enhance lipid peroxidation in the plasma and mitochondrial membranes and lead to the aggregation of cytotoxic aldehydes, such as 4-HNE [37, 38]. Previous studies have revealed that ROS and 4-HNE collectively play a fundamental role in initiating immune defense and participating in the inflammatory process and cell death signaling pathways [39–41]. In a murine model of LPS-induced cardiac injury, ALDH2 OE or activation alleviated cardiac injury and cardiomyocyte apoptosis by mitigating ROS and 4-HNE accumulation [16]. Similarly, the present study showed that ROS, 4-HNE, and apoptosis levels were upregulated after LPS administration. In addition, defects in ALDH2 aggravate ROS, 4-HNE, and apoptosis in septic AKI. Thus, ALDH2 might be closely related to the pathogenesis of septic AKI; however, the underlying mechanisms of its role need to be delineated.

The MAPK pathway is one of the most important signal transduction pathways in cell proliferation, inflammation, differentiation, and apoptosis [42]. Recent studies have emphasized that activation of the MAPK pathway is involved in regulating inflammatory responses in sepsis-induced organ dysfunction [43, 44]. In addition, ROS generation and 4-HNE accumulation are both efficient mediators of MAPK pathway activation [45–47]. Previous studies indicate that upregulation of ALDH2 prevents acetaldehyde-induced cell injury by inhibiting ROS generation and suppressing the ERK and p38 MAPK pathways [48]. Conversely, inhibition of ALDH2 activity by daidzin enhances antimycin-induced cardiomyocyte apoptosis via MAPK activation [49]. The results of our study indicated an increase in the phosphorylation of JNK and p38 after LPS treatment. Interestingly, phosphorylation of JNK and p38 decreased following ALDH2 OE; however, the levels of phosphorylated p38, but not those of phosphorylated JNK, were augmented after ALDH2 KD following the LPS treatment of TECs. Based on the results of our study, it can be speculated that LPS-induced renal tubular cell apoptosis is mediated, at least in part, by enhanced ROS generation and 4-HNE accumulation, followed by the activation of the MAPK pathway, that is, of JNK and p38 MAPK. Our observations suggest that the MAPK pathway is pivotal for ALDH2-induced renal protection during septic AKI.

Alda-1, a specific agonist of mitochondrial ALDH2, has been developed as an effective approach to preventing or eliminating excessive aldehyde production [18, 50]. Studies have demonstrated the protective effects of Alda-1 in various settings [14, 15, 17]. Alda-1 administration prior to ischemia ameliorates ischemia-reperfusion injury-induced renal pathological damage via upregulation of ALDH2 enzymic activity [51]. Nevertheless, prolonged infusion of Alda-1 following renal ischemia in rats resulted in intratubular crystal deposition in the kidneys along with the deterioration of renal function, suggestive of crystalline nephropathy [52]. In the present study, after pretreatment with Alda-1 before LPS administration, we observed that kidney pathological injury and decline in renal function were alleviated, ROS and 4-HNE levels were decreased, inflammatory reaction was inhibited, and apoptosis was improved. A possible explanation for these discrepancies in Alda-1 effects may be the different intervention periods and experimental settings. Further studies are required to determine the optimal dose and duration of Alda-1 treatment under various AKI settings. Taken together, these results indicate that ALDH2 activation by Alda-1 pretreatment may provide a promising option for the prevention or treatment of septic AKI.

**Figure 7:**
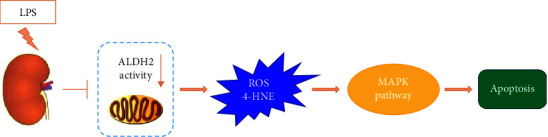
Proposed schema of effect of ALDH2 on the LPS-induced septic AKI, involving the inhibition of ALDH2 activity, promotion of ROS and 4-HNE generation, upregulation of MAPK pathway, and resulting in renal cell apoptosis.

In summary, our results suggest that LPS administration suppresses ALDH2 activity in a septic AKI mouse model, leading to ROS generation and 4-HNE accumulation. The increased levels of ROS and 4-HNE may enhance the inflammatory response and apoptosis through the MAPK-dependent pathway and further promote septic AKI progression (Figure 7). Upregulating ALDH2 expression or enhancing ALDH2 activity may emerge as a promising therapeutic strategy for septic AKI.

## Figures and Tables

**Table 1:** PCR primer sequences.

Gene	Forward primer	Reverse primer
TNF-*α*	GCCTCTTCTCATTCCTGCTTGT	TTGAGATCCATGCCGTTG
IL-6	GCTACCAAACTGGATATAATCAGGA	CCAGGTAGCTATGGTACTCCAGAA
IL-10	ACTGCACCCACTTCCCAGT	TGTCCAGCTGGTCCTTTGTT
*β*-Actin	GATTACTGCCCTGGCTCCTA	TCATCGTACTCCTGCTTGCT

## Data Availability

The datasets are available from the corresponding authors on reasonable request.

## Supplementary Materials

Supplementary Materials Figure S1: Alda-1 pretreatment reduced the LPS-induced inflammatory response. Figure S2: The role of ALDH2 on the 4-HNE expression in TECs. Figure S3: Role of ALDH2 on the ROS generation in tubular epithelial cells (TECs). Figure S4: Alda-1 pretreatment alleviated LPS-induced apoptosis in the kidneys. Figure S5: Alda-1 pretreatment improved LPS-induced apoptosis in TECs.
