# Supplementary material for: Aldehyde Dehydrogenase 2 Ameliorates LPS-Induced Acute Kidney Injury through Detoxification of 4-HNE and Suppression of the MAPK Pathway
Source: J Immunol Res. 2023 Apr 6;2023:5513507. doi: 10.1155/2023/5513507 (PMC10101750; doi:10.1155/2023/5513507)
Supplement: Supplementary Materials — Figure S1: Alda-1 pretreatment reduced the LPS-induced inflammatory response. Figure S2: The role of ALDH2 on the 4-HNE expression in TECs. Figure S3: Role of ALDH2 on the ROS generation in tubular epithelial cells (TECs). Figure S4: Alda-1 pretreatment alleviated LPS-induced apoptosis in the kidneys. Figure S5: Alda-1 pretreatment improved LPS-induced apoptosis in TECs. [file 5513507.f1.docx]

**Supplementary Figures**


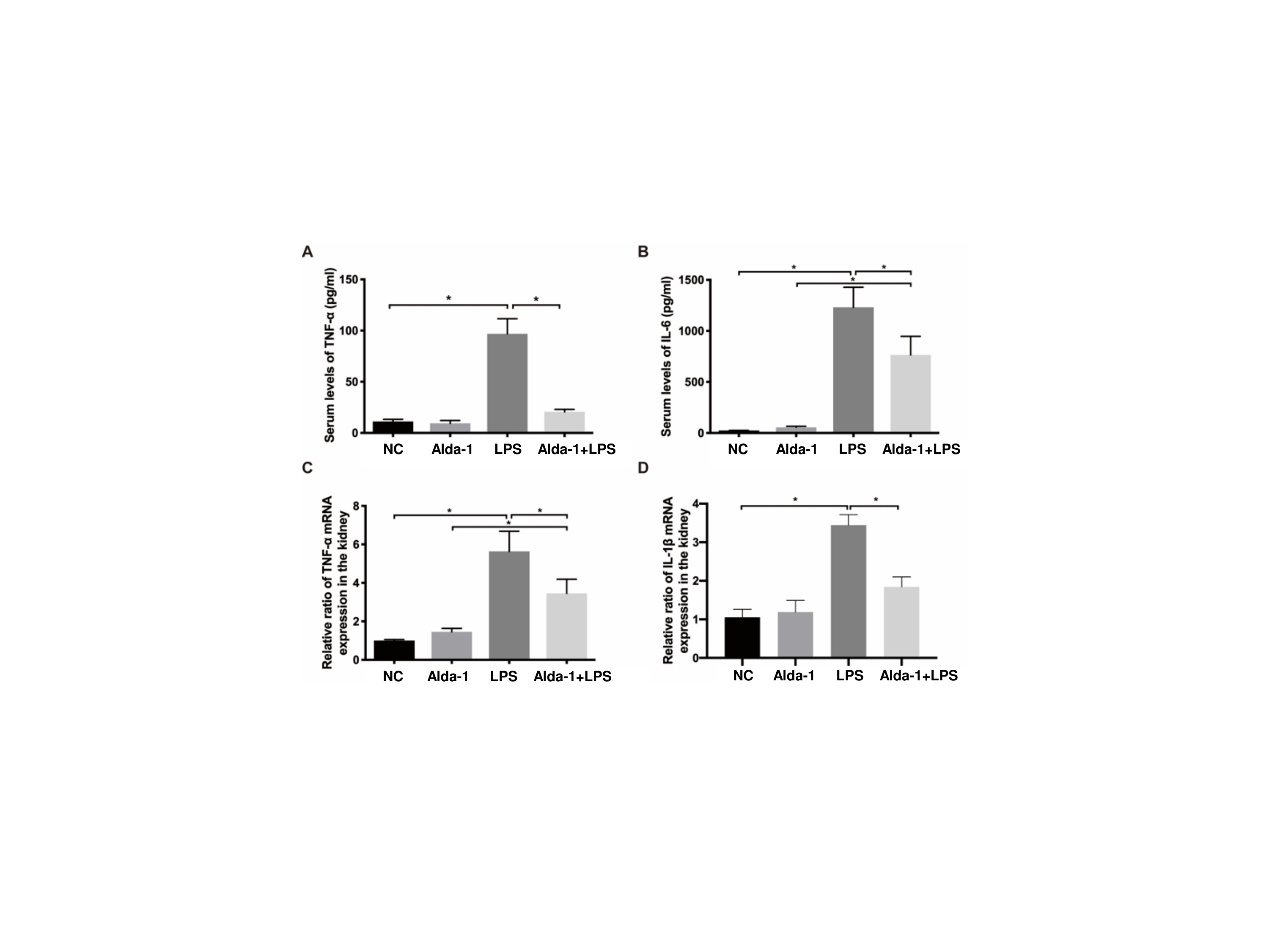


**Supplementary Figure 1.** Alda-1 pretreatment reduced the LPS-induced inflammatory response. **(A, B)** The protein levels of TNF-α and IL-6 were measured by ELISA in the kidney homogenate. **(C, D)** The mRNA levels of TNF-α and IL-1β were measured by PCR analysis in the kidney homogenate. All data are expressed as mean ± SEM (*n* = 6). **P* < 0.05.


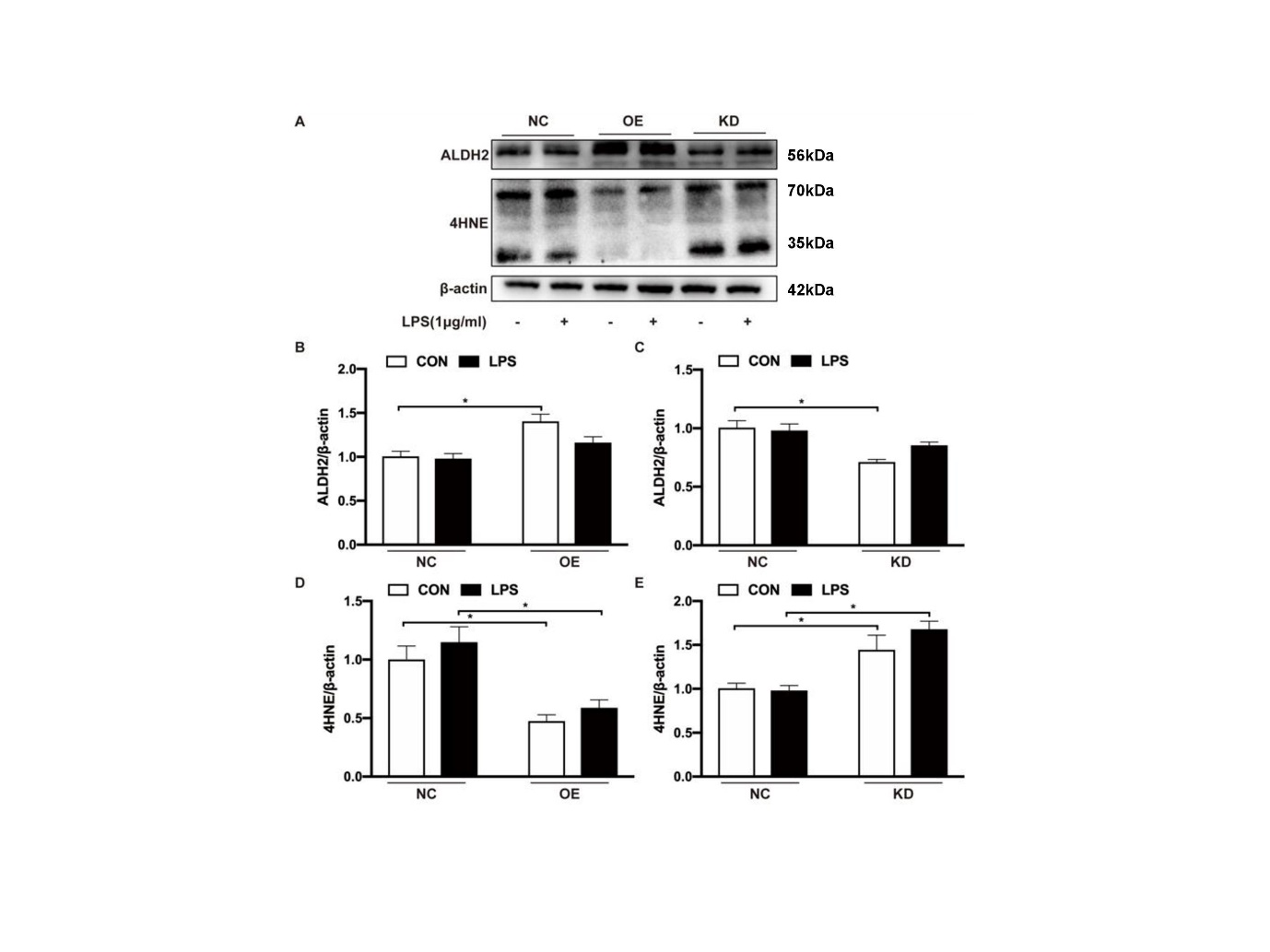


**Supplementary Figure 2.** The role of ALDH2 on the 4-HNE expression in TECs. **(A)** Representative western blots of ALDH2, 4-HNE, and β-actin (loading control) in OE, KD, and NC cells after the LPS challenge. **(B, C)** Quantification analysis of ALDH2 expression NC, OE, and KD cells. **(D, E)** Quantification analysis of 4-HNE expression in NC, OE, and KD cells. All data are expressed as mean ± SEM (*n* = 6). **P* < 0.05.


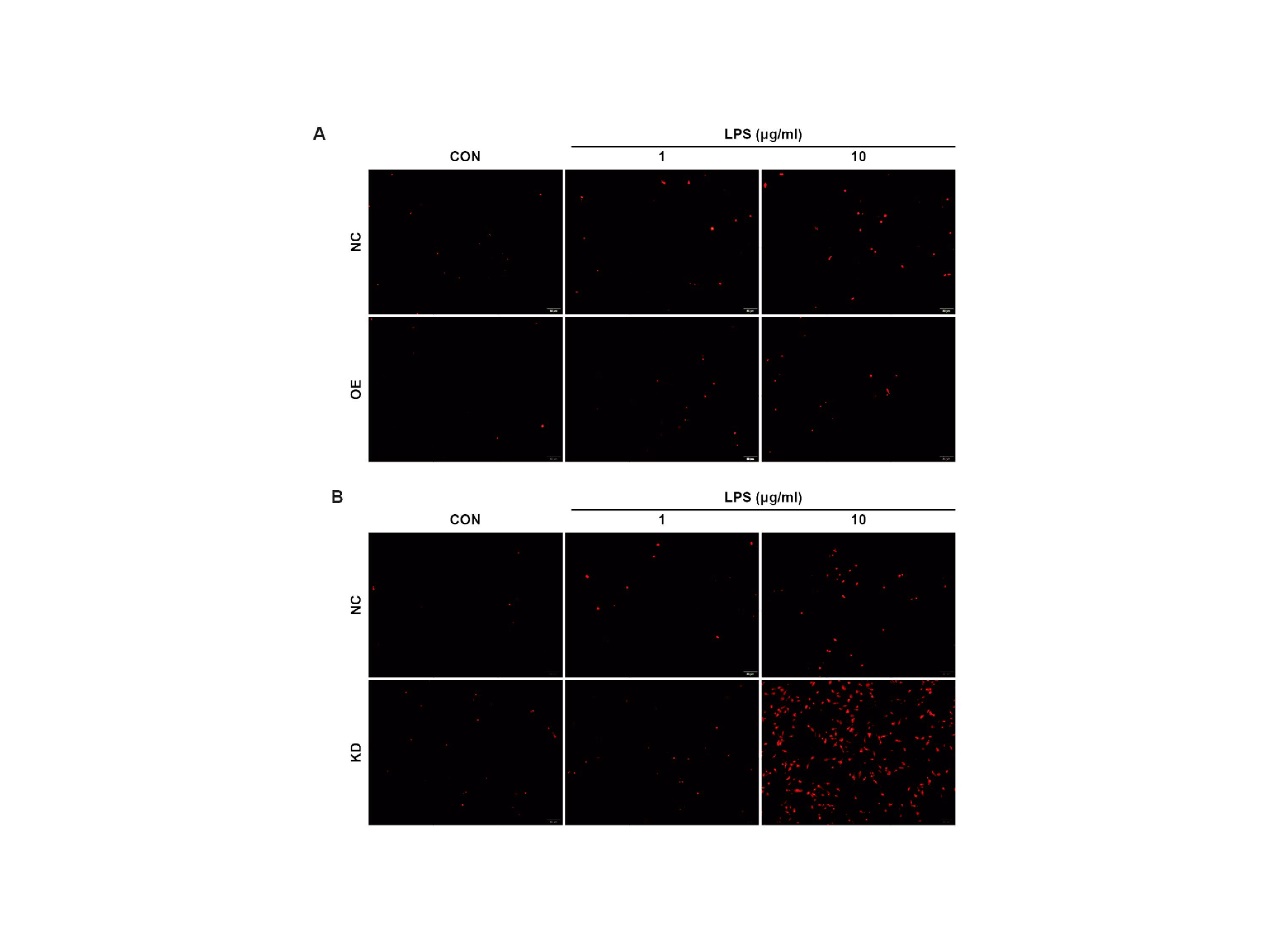


**Supplementary Figure 3.** Role of ALDH2 on the ROS generation in tubular epithelial cells (TECs). **(A)** ROS expression in OE and NC cells after the LPS challenge (1 μg/ml and 10 μg/ml, respectively). **(B)** ROS expression in KD and NC cells after the LPS challenge (1 μg/ml and 10 μg/ml, respectively). Scale bars: 50 μm.

**
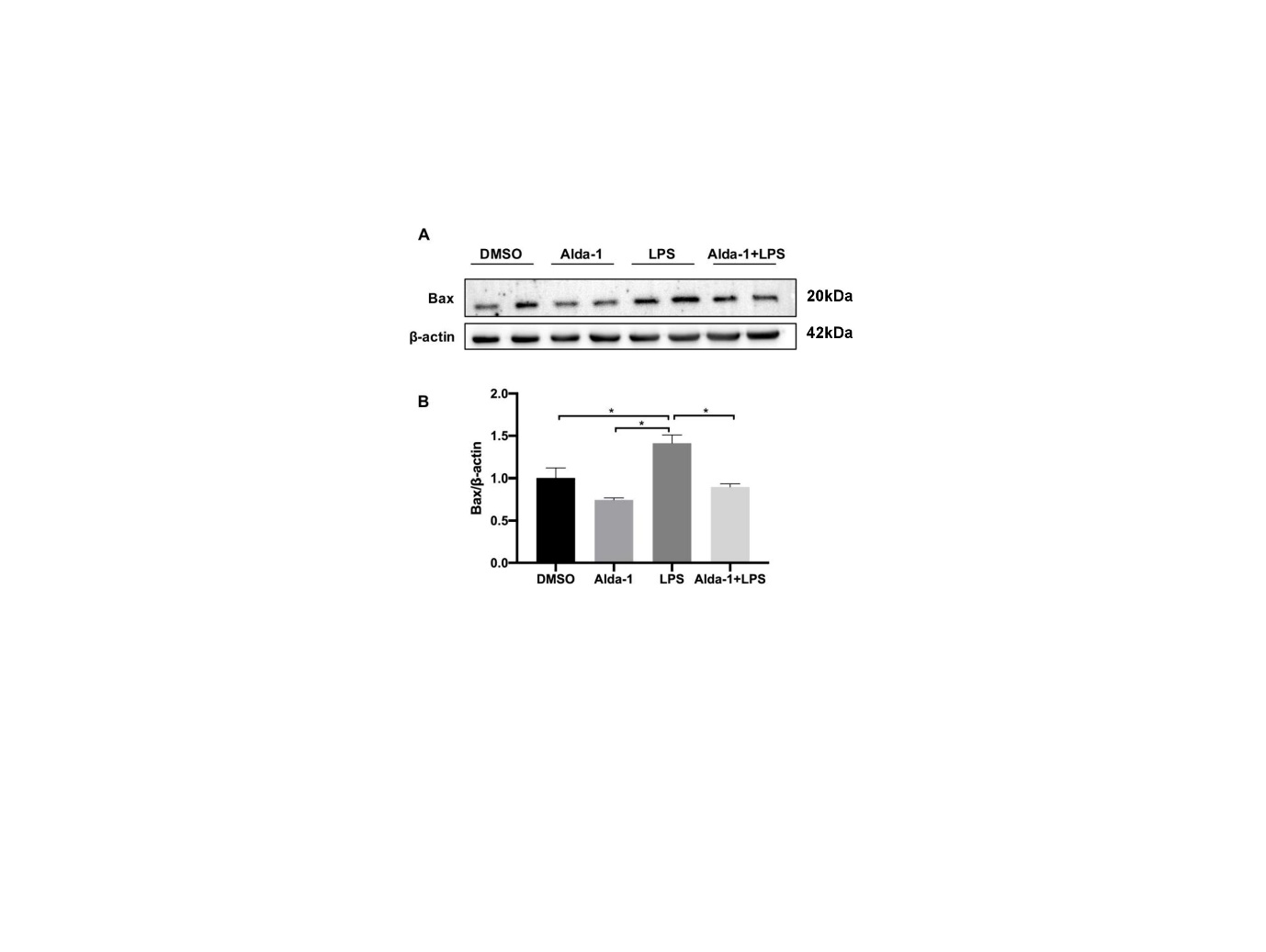
**

**Supplementary Figure 4.** Alda-1 pretreatment alleviated LPS-induced apoptosis in the kidneys. **(A)** Representative western blots of Bax and β-actin (loading control) in mice with or without Alda-1 pretreatment. **(B)** Quantification analysis of Bax expression in the kidneys of mice with or without Alda-1 pretreatment. All data are expressed as mean ± SEM (*n* = 6). **P* < 0.05.


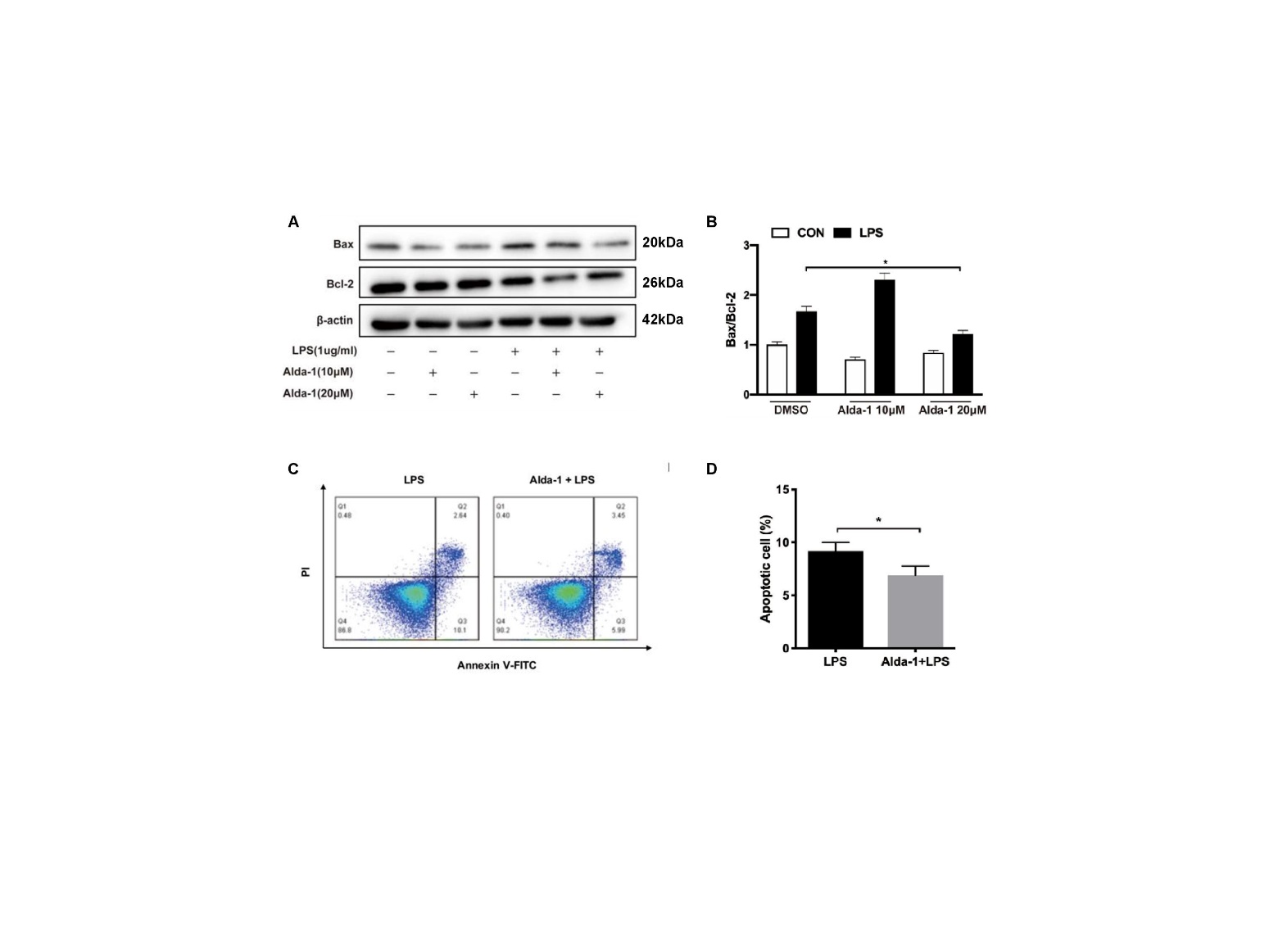


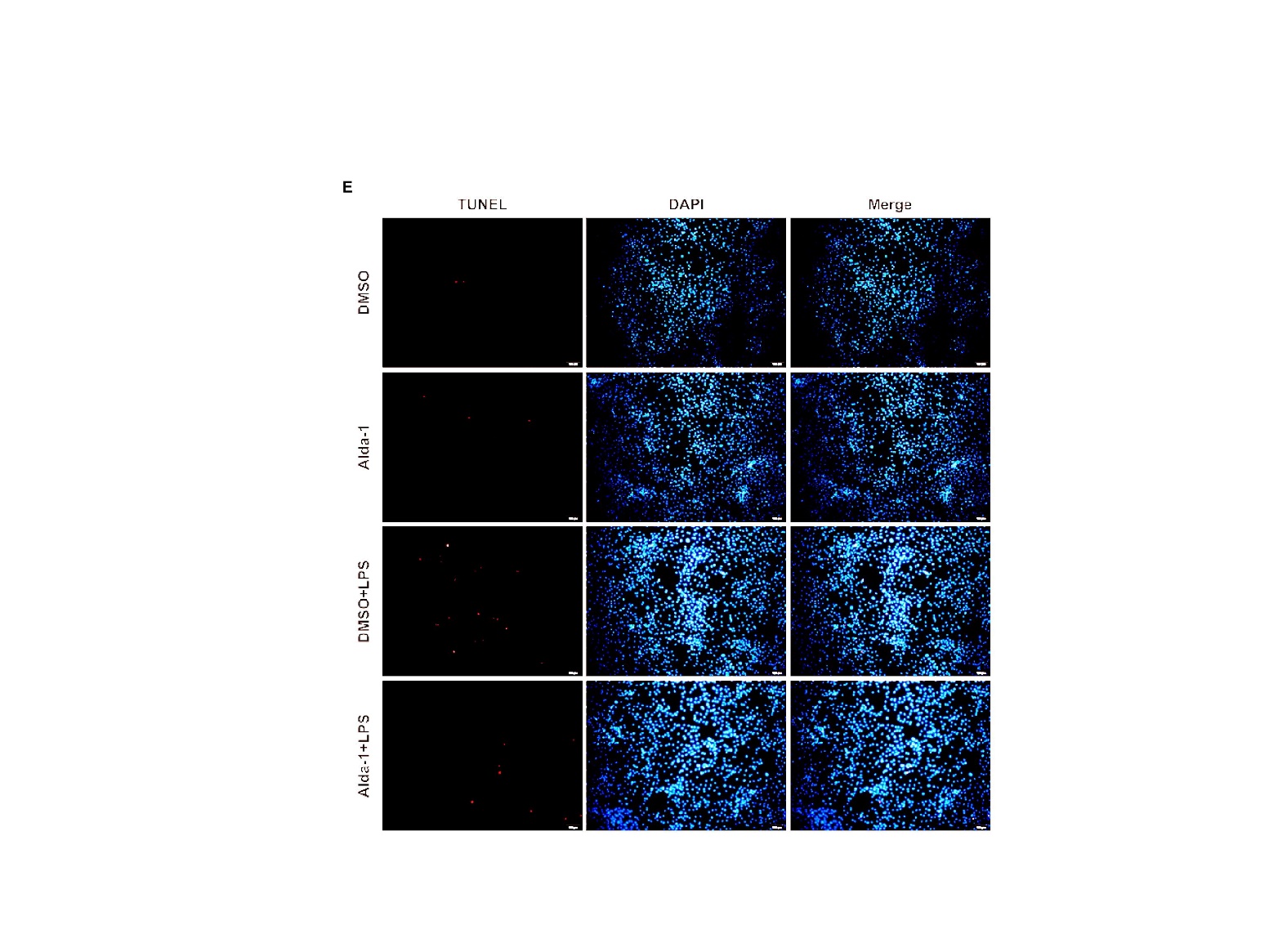


**Supplementary Figure 5.** Alda-1 pretreatment improved LPS-induced apoptosis in TECs. **(A, B)** Representative western blots and quantification analysis of Bax, Bcl2, and β-actin (loading control) in TECs with or without Alda-1 pretreatment. **(C, D)** Flow cytometric and quantification analysis of apoptosis in TECs with or without Alda-1 pretreatment. **(E)** Representative images of the TUNEL assay in TECs after the LPS challenge. Scale bars: 50 μm. All data are expressed as mean ± SEM (*n* = 6). **P* < 0.05.
